# Evaluation of [^18^F]MK-6240 binding to tau protein in postmortem human brains of Down syndrome and Alzheimer’s disease and assessment of off-target (non-tau) binding

**DOI:** 10.21203/rs.3.rs-8463304/v1

**Published:** 2026-01-06

**Authors:** Fariha Karim, Agnes P. Biju, Christopher Liang, Camryn J. Santos, Maharishi Rajarethenam, Jogeshwar Mukherjee

**Affiliations:** University of California Irvine; University of California Irvine; University of California Irvine; University of California Irvine; University of California Irvine; University of California Irvine

**Keywords:** Down Syndrome, Alzheimer’s disease, Tau, Imaging, Harmine, Meninges

## Abstract

Alzheimer’s disease (AD) and Down Syndrome (DS) are characterized by the aggregation of tau tangles. As a novel tau PET tracer in AD, [^18^F]MK-6240 has the potential in DSAD to elucidate pathophysiology and advance diagnostic strategies. Autoradiography of frontal cortex (FCX) and temporal cortex (TCX) postmortem brain slices of DSAD (n = 5), AD (n = 5), and cognitively normal (CN) (n = 5) cases indicated similarly high [^18^F]MK-6240 binding in DSAD and AD cases. Anti-tau immunostains confirmed total tau presence so there was alignment in anti-tau abundance with quantification of [^18^F]MK-6240 binding. DSAD and AD cases exhibited higher gray matter (GM)/white matter (WM) ratios of 2.8 and 2.5 respectively. For drug effects on [^18^F]MK-6240 binding, self-displacement of [^18^F]MK-6240 was by 88% among DSAD cases and 85% among AD cases while IPPI displaced [^18^F]MK-6240 by 81% and 74% in DSAD and AD cases respectively. KuFal194, a specific phosphokinase inhibitor, minimally displaced [^18^F]MK-6240 binding. Harmine competed with [^18^F]MK-6240 with an IC_50_ value of 290 ± 218 nM and 92 ± 15 nM for DSAD and AD cases, respectively, suggesting unique tau binding. High meninges off-target (non-tau) binding of [^18^F]MK-6240 was observed in a CN case, comparable to the GM in DSAD and AD. MK-6240 (10 μM) blocked 44% and T807 (10 μM) blocked 30% of meninges binding. Incubation of meninges in the presence of 0.2% polyethylenimine reduced 70% of [^18^F]MK-6240 binding. The tau imaging agent, [^125^I]IPPI, an analog of [^18^F]MK-6240, exhibited minimal binding to CN meninges. Our findings suggest [^18^F]MK-6240 to be selective tau imaging agent in DSAD and AD, harmine to be a weak tau drug, and off-target nonspecific meninges binding maybe due to the primary aromatic amine group in [^18^F]MK-6240.

## 1. Introduction

Adults with Down syndrome (DS) are predisposed to develop Alzheimer’s disease (AD) starting with its pathology of fibrillar amyloid beta (Aβ) plaque and neurofibrillary tangles [41, 47, 58]. The pathological correlation between DS and AD may be affected by the triplication of chromosome 21 in DS, causing an abundance of Aβ plaques and neurofibrillary tangles. The deposition of tau closely parallels the severity of cognitive decline in AD more than Aβ plaque, emphasizing tau mechanisms to track disease progression [8, 40]. The downregulation of protein phosphatase 2A, a major tau regulator, results in abnormal tau hyperphosphorylation to disrupt protein homeostasis via oxidative damage and energy metabolic dysfunction [10, 30, 45]. Neurofibrillary tangles arise from the abnormal hyperphosphorylation of normal tau, leading to neurofibrillary degeneration that contribute to cognitive impairment in AD [21, 57]. Tau accumulation is similar in DS and AD throughout the brain originating from the entorhinal region to gradually spread into the limbic regions and eventually the isocortical regions [7, 46]. The frontal cortex (FCX) is suggested as an early region for AD pathology to develop in DS, possibly underlying executive dysfunction and behavioral changes that align with the rate of cognitive deterioration [1, 19, 27]. Dual specificity tyrosine-phosphorylation regulated kinase 1A (DYRK1A) promotes tau phosphorylation and is overexpressed in DS, further contributing to neurofibrillary degeneration that may develop into AD pathogenesis [32, 49].

Positron emission tomography (PET) studies using tau imaging agents provide visual opportunities to improve upon diagnostic strategies in multiple diseases including AD. [^18^F]flortaucipir (also known as T807) was among the first tau PET tracers to show promise in evaluations of clinical AD with its strong affinity to tau aggregates [13, 35, 59]. However, off-target binding and low tau affinity in non-AD dementias are limitations of [^18^F]flortaucipir [34, 36, 51]. Second generation tau PET tracers sought to improve upon the capabilities of [^18^F]flortaucipir such as [^18^F]MK-6240 which has been validated throughout multiple in vivo and in vitro studies [2, 3, 20]. The advantages of [^18^F]MK-6240 include higher affinity to tau aggregates, more sensitive detection of tau in early AD progression, and greater dynamic range in standardized uptake value ratio (SUVR) estimates [4, 20]. Although [^18^F]MK-6240 improves upon some features of [^18^F]flortaucipir, [^18^F]MK-6240 also exhibits different off-target, nonspecific binding such as binding to the meninges in a number of PET studies [2, 33]. More thorough investigations of [^18^F]MK-6240 and its binding characteristics in various conditions are necessary, especially when information about [^18^F]MK-6240 and other tau PET tracers in DSAD is currently limited. Significant binding of [^18^F]MK-6240 was demonstrated in genetic frontotemporal dementia but not in other non-AD tauopathies such as progressive supranuclear palsy and Pick’s disease [2, 28].

To continue the efforts to improve upon existing tau radiotracers, we have developed several azaindole derivatives, such as IPPI [39], INFT [31], and ISAS [52], to use on postmortem AD brain slices to uncover their capabilities. Recently, [^125^I]IPPI effectively displayed high selectivity for tau in not only AD cases but also in DSAD cases, showing promise as an analog of [^18^F]MK-6240 [5, 23]. The success of [^18^F]MK-6240 in AD cases implicates potential as a tau imaging agent in DSAD [22]. Since the FCX is an early area of AD pathology in DS and tau accumulation initially spreads to limbic regions including the temporal cortex (TCX), it is of interest to study the FCX and TCX for similarities in [^18^F]MK-6240 binding [27, 46–47]. This comparative autoradiography study using [^18^F]MK-6240 in DSAD, AD, and cognitively normal (CN) cases can augment understanding of tau accumulation and its pathophysiological attributes in human FCX and TCX. The binding characteristics of [^18^F]MK-6240 may also be revealed when observing drug effects and off-target binding to meninges including comparisons to [^125^I]IPPI.

## 2. Materials and Methods

### 2.1. Human Tissue Samples

Human postmortem brain tissue samples of DSAD, AD, and CN cases (male and female), each consisting of FCX and TCX, were obtained from UCI Memory Impairment and Neurological Disorders (MIND) institute for in vitro experiments (Table 1). Brain slices, 10 μm thick, were obtained from frozen tissue using a Leica 1850 cryotome cooled to −20 °C and collected on Fisher glass slides. All slides were then stored at −80 °C. Frozen cerebral meninges glass-mounted tissue slices (each slide containing two 10 μm thick slices) from adult normal subject, NBP2–77524, batch A610385 were purchased from Novus Biologicals, Colorado, USA. Meninges slices were hematoxylin and eosin (H&E) stained as well as stained with anti-tau as described in section 2.3. All postmortem human brain studies were approved by the Institutional Biosafety Committee of University of California, Irvine.

### 2.2. Radiosynthesis

Radiosynthesis of [^18^F]MK-6240 was carried out using modifications of reported procedures [15, 55]. High specific activity fluorine-18 radioactivity was obtained from PETNET, Inc (Culver City, CA) and counted in a Capintec CRC-15R dose calibrator. This hydrogen [^18^F]fluoride in H_2_^18^O was passed through light QMA Sep-Pak (Waters Corp.), preconditioned with 2 mL of potassium carbonate, 140 mg/mL, followed by 2 mL of anhydrous acetonitrile. The trapped [^18^F]fluoride in QMA was eluted with 2.5 mL of Kryptofix 2.2.2 (Aldrich)/potassium carbonate solution (36 mg Kryptofix and 7.5 mg potassium carbonate in 2.4 mL acetonitrile and 0.1 mL water) and transferred to the reaction vessel. The initial drying step of the [^18^F]fluoride, Kryptofix 2.2.2., and K_2_CO_3_ mixture was at 120°C for 10 min under a stream of nitrogen gas. The [^18^F]fluoride solution was further dried with acetonitrile (2×1 mL) at 120 °C for 7 min. To the dried [^18^F]fluoride reaction mixture, [^18^F]MK-6240 precursor, *N*-[(tert-butoxy)carbonyl-*N*-(6-nitro-3-[1H-pyrrolo[2,3-c]pyridine-1-yl)isoquinolin-5-yl)carbamate (1ClickChemistry, Inc., New Jersy, USA) 2 mg dissolved in 1 mL dimethylformamide (DMF) was added. This reaction mixture was heated at 160 °C for 20 min and then cooled. Methanol (5 mL) was added to the reaction vial and the contents were passed through neutral alumina Sep-Paks (Waters, Inc), prewashed with methanol. The DMF-methanolic containing [^18^F]MK-6240 was evaporated *in vacuo* followed by semipreparative HPLC purification using an Alltech C_18_ column (10 μm, 250×10 mm) and UV detector (254 nm), mobile phase: 60% acetonitrile-40% 0.1% aqueous triethylamine, 2.5 mL/min, r.t. = 22 min. [^18^F]MK-6240 was made in modest yields (30% decay corrected) in specific activities of 74 GBq/μmol. The collected fraction was taken to near dryness *in vacuo* and final product was taken up in 10% alcohol in sterile saline.

Radiosynthesis of [^125^I]IPPI was carried out as previously reported [39].

### 2.3. Immunohistochemistry

Adjacent brain slices of all cases and meninges were immunostained for tau by UCI Pathology core services. For total Tau, DAKO polyclonal antibody which detects all 6 six isoforms of tau from the microtubule-associated protein tau gene, was used at a dilution 1:3000, A0024 (Agilent, CA, USA) using reported protocols [17]. Adjacent slices of meninges and 08–42 DSAD FCX were stained with H&E to compare with each other. Immunostained slides were scanned using the Ventana Roche slide scanner and the images generated were used for analysis by QuPath (version 0.4.4). Several annotations for tau and negative annotations with no tau were made on the immunohistochemistry (IHC) images of the brain slices for each case. After training on the manual annotations, a pixel classifier was run on the entire brain slice to visualize where the tau was and was saved as an image.

### 2.4. [^18^F]MK-6240 Autoradiography

Brain sections and meninges were treated with approximately 10 μCi/mL [^18^F]MK-6240 (407 kBq/mL) in phosphate buffered saline (PBS) pH 7.4. The chambers were incubated at 25 °C for 1 hour and then underwent multiple washes of 100% PBS for 1 minute, 70% ethanol in PBS for 2 minutes, 30% ethanol in PBS for 1 minute, and lastly 100% PBS for 1 minute [2]. The brain sections were air dried and then transferred into a film cassette with a phosphor screen film. After 24 hours, the films were taken out of the cassette and were read on the Phosphor Autoradiographic Imaging System/Cyclone Storage Phosphor System (Packard Instruments Co). The acquisition and analysis program OptiQuant (Packard Instruments Co, version 5.0) was used to draw regions of interest (ROIs) on the autoradiographic images and the extent of binding was measured in digital light unit per unit mm^2^ (DLU/mm^2^). Background activity levels were subtracted from all images. Higher DLU/mm^2^ from autoradiography indicated higher [^18^F]MK-6240 binding.

### 2.5. Autoradiography of In Vitro Drug Inhibition on [^18^F]MK-6240

Inhibitor concentration (IC_50_) of harmine on [^18^F]MK-6240 binding was determined by using adjacent brain slices of FCX and TCX DSAD cases with different concentrations (10^− 9^ to 10^− 5^M) of harmine (Adooq Bioscience, Irvine, CA). Unlabeled MK-6240 (1ClickChemistry, Inc, Tinton Falls, New Jersey, USA) at 10 μM was designated for nonspecific binding to determine specific binding of [^18^F]MK-6240. Binding studies used 407 kBq/mL of [^18^F]MK-6240 per cc of ethanol with incubation at 25°C for 1 hour. Then the slices underwent washes consisting of 100% PBS for 1 minute, 70% ethanol in PBS for 2 minutes, 30% ethanol in PBS for 1 minute, and lastly 100% PBS for 1 minute. Brain sections were air dried, exposed to a phosphor screen film for 48 hours, then the film was placed on the Phosphor Autoradiographic Imaging System/Cyclone Storage Phosphor System (Packard Instruments Co). OptiQuant was used to draw ROIs on autoradiographic images and [^18^F]MK-6240 binding was measured in DLU/mm^2^. Nonspecific binding was subtracted from the total binding to calculate specific binding at different concentrations of harmine. Inhibitor concentration, IC_50_ was measured by plotting specific binding against harmine concentrations using GraphPad Prism 10.

To investigate drug effects, unlabeled MK-6240, IPPI, and KuFal194 (10-iodo-11H-indolo[3,2-c]quinoline-6-carboxylic acid) (AABlocks LLC, San Diego, CA, USA) were used with [^18^F]MK-6240 binding to adjacent slices of FCX and TCX DSAD cases. The incubation of [^18^F]MK-6240 in the total binding and competition experiments with KuFal194 (10 μM) included 10 μM of the urea, 1,3-bis(4-cyanophenyl)urea (BCU) (1ClickChemistry, Inc, Tinton Falls, New Jersey, USA) as previously reported [42]. Inclusion of BCU has been shown to assist in membrane permeability of carboxylic acid containing drugs such as KuFal194 [50]. Incubation and washing procedures were the same as the other experiments in this study.

To investigate [^18^F]MK-6240 binding to meninges, total binding of [^18^F]MK-6240 to meninges slices was carried out. Additionally, [^18^F]MK-6240 binding in the presence of unlabeled MK-6240 (10 μM) for self-displacement and T807 (10 μM) (AABlocks LLC, San Diego, CA, USA) separately. Potential effects of reducing nonspecific binding by inclusion of 0.2% polyethylenimine (PEI, Sigma-Aldrich) in PBS incubation buffer was also investigated. [^125^I]IPPI (10 μCi/mL) was also evaluated for binding to meninges along with other AD and DSAD FCX and TCX cases under normal PBS conditions and with PBS with 0.2% PEI. Incubation and washing procedures were the same as the other experiments in this study. Brain tissue treated with [^125^I]IPPI exposed to a phosphor screen film for 2 weeks before reading the film.

### 2.6. Statistical Analysis

The DLU/mm^2^ values from OptiQuant were analyzed in GraphPad Prism 10 and Microsoft Excel 16. Student’s t-test were performed with p values < 0.05 indicating statistical significance. Error bars are presented as mean ± standard deviation. The Shapiro-Wilk test confirmed normality of distribution among all groups. Pearson’s correlation and linear regression was used for the parametric comparison between [^18^F]MK-6240 and [^125^I]IPPI DLU/mm^2^ values [5] within DSAD and AD cases were determined with Pearson’s correlation and linear regression in GraphPad Prism 10. Post hoc power analysis (Prism, version 10) with specified means and alpha = 0.05 indicated > 90% power for n = 5 DSAD and > 60% power for n = 5 AD cases when compared to CN cases in both FCX and TCX. Since [^18^F]MK-6240 has already been established in AD, greater power was prioritized in DSAD.

## 3. Results

### 3.1 [^18^F]MK-6240 Binding in DSAD, AD, and CN Cases

#### 3.1.1. DSAD Cases

**Figure 1:**
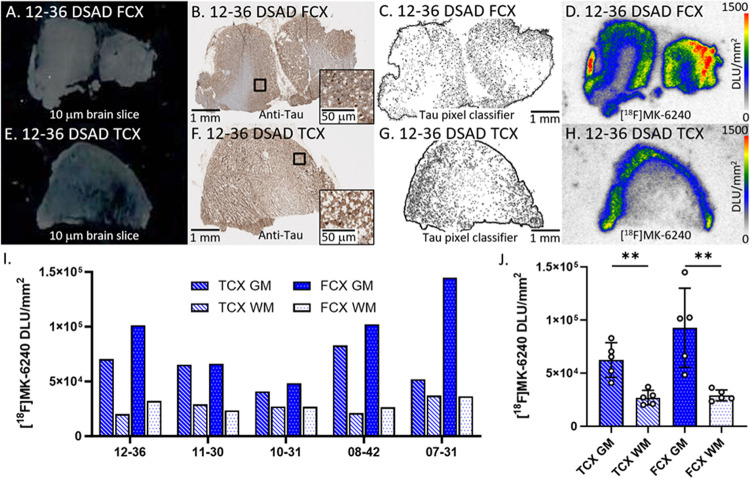
[^18^F]MK-6240 binding to tau in DSAD: **(A)**. Postmortem human brain slice (10 μm) of DSAD FCX 12–36. **(B)**. Anti-tau IHC of DSAD FCX 12–36 adjacent brain slice, inset of 50 μm magnification. **(C)**. Tau pixel classifier image of DSAD FCX 12–36. **(D)**. [^18^F]MK-6240 binding to DSAD FCX 12–36; autoradiography scale bar: 0–1500 DLU/mm^2^. **(E)**. Postmortem human brain slice (10 μm) of DSAD TCX 12–36. **(F)**. Anti-tau IHC of DSAD TCX 12–36 adjacent brain slice, inset of 50 μm magnification. **(G)**. Tau pixel classifier image of DSAD TCX 12–36. **(H)**. [^18^F]MK-6240 binding to DSAD TCX 12–36; autoradiography scale bar: 0–1500 DLU/mm^2^. **(I)**. [^18^F]MK-6240 binding in DLU/mm^2^ to all DSAD cases within GM and WM of TCX and FCX regions. **(J)**. Average [^18^F]MK-6240 binding to all DSAD cases separated by TCX and FCX GM and WM regions (** p<0.001).

Binding of [^18^F]MK-6240 was evaluated in all DSAD cases (Fig. 1). Fig. 1A–D showed adjacent slices of representative case 12–36 DSAD FCX while Fig. 1E–H showed adjacent slices of case 12–36 DSAD TCX. Within adjacent brain slices of all cases, the presence of tau was confirmed by the anti-tau immunostainings (Fig. 1B, 1F). The tau pixel classifier confirmed the abundance of tau mostly within the gray matter (GM) regions (Fig. 1C, 1G). The areas on the slice where there was anti-tau detected and [^18^F]MK-6240 binding on autoradiographic images aligned with each other. There was substantially more [^18^F]MK-6240 binding in GM than white matter (WM) among both TCX and FCX regions (Fig. 1I, 1J). The extent of GM binding of [^18^F]MK-6240 varied across the subjects in both the brain regions with the FCX showing higher average GM compared to TCX (Fig-1J). The average GM/WM ratio in TCX was 2.5 while FCX had a GM/WM ratio of 3.1 in DSAD.

#### 3.1.2. AD Cases

**Figure 2:**
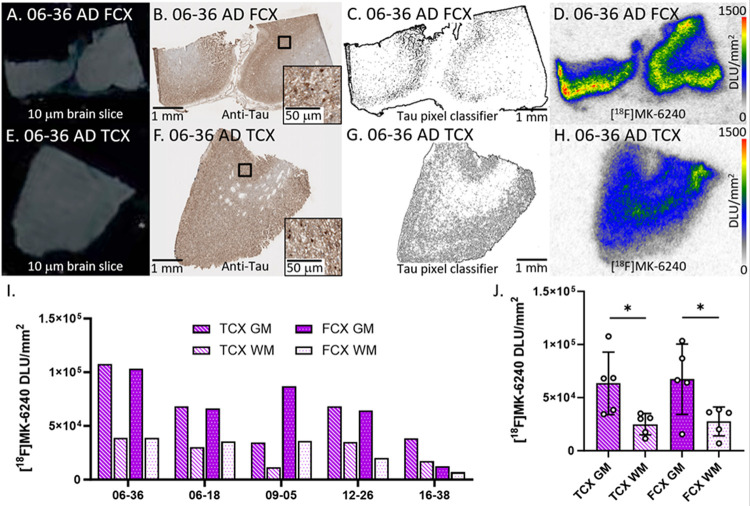
[^18^F]MK-6240 binding to tau in AD: **(A)**. Postmortem human brain slice (10 μm) of AD FCX 06–36 **(B)**. Anti-tau IHC of AD FCX 06–36 adjacent brain slice, inset of 50 μm magnification. **(C)**. Tau pixel classifier image of AD FCX 06–36. **(D)**. [^18^F]MK-6240 binding to AD FCX 06–36; autoradiography scale bar: 0–1500 DLU/mm^2^. **(E)**. Postmortem human brain slice (10 μm) of AD TCX 06–36. **(F)**. Anti-tau IHC of AD TCX 06–36 adjacent brain slice, inset of 50 μm magnification. **(G)**. Tau pixel classifier image of AD TCX 06–36. **(H)**. [^18^F]MK-6240 binding to AD TCX 06–36; autoradiography scale bar: 0–1500 DLU/mm^2^. **(I)**. [^18^F]MK-6240 binding in DLU/mm^2^ to all AD cases within GM and WM of TCX and FCX regions. **(J)**. Average [^18^F]MK-6240 binding to all AD cases separated by TCX and FCX GM and WM regions (* p<0.05).

The [^18^F]MK-6240 binding in all AD cases were evaluated (Fig. 2). Fig. 2A–D displayed adjacent slices of representative case 06–36 AD FCX while Fig. 2E–H displayed 06–36 AD TCX. Anti-tau immunostains (Fig. 2B, 2F) of the five AD cases revealed the presence of tau, confirmed by the tau pixel classifier (Fig. 2C, 2G). There was general alignment between the amount of anti-tau detected and the [^18^F]MK-6240 binding on AD TCX autoradiographic images. The [^18^F]MK-6240 binding in TCX and FCX cases were greater in GM compared to WM (Fig. 2I, 2J). Variation in the GM binding of [^18^F]MK-6240 across the subjects was observed in both the brain regions with a similar average level of binding in both the FCX and TCX (Fig-2J). The average GM/WM ratio in TCX was 2.4 while FCX had a GM/WM ratio of 2.5 in AD.

#### 3.1.3. CN Cases

**Figure 3:**
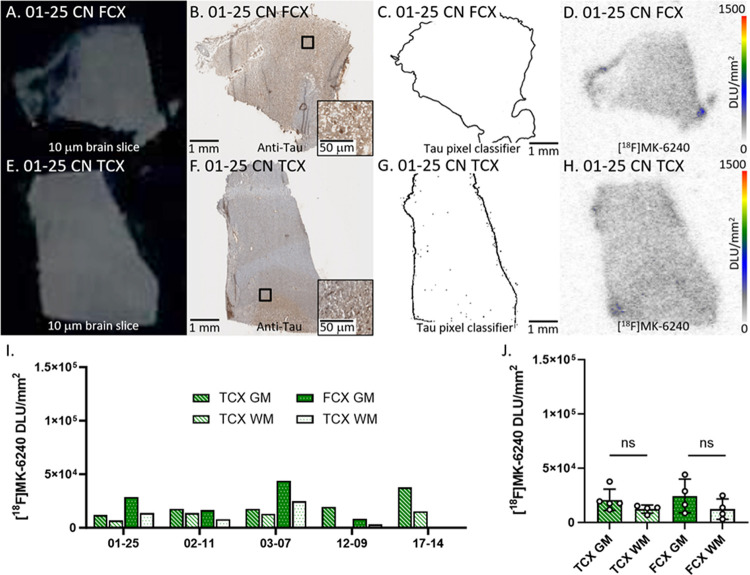
[^18^F]MK-6240 binding to tau in CN: **(A)**. Postmortem human brain slice (10 μm) of CN FCX 01–25. **(B)**. Anti-tau IHC of CN FCX 01–25 adjacent brain slice, inset of 50 μm magnification **(C)**. Tau pixel classifier image of CN FCX 01–25 **(D)**. [^18^F]MK-6240 binding to CN FCX 01–25; autoradiography scale bar: 0–1500 DLU/mm^2^. **(E)**. Postmortem human brain slice (10 μm) of CN TCX 01–25. **(F)**. Anti-tau IHC of CN TCX 01–25 adjacent brain slice, inset of 50 μm magnification. **(G)**. Tau pixel classifier image of CN TCX 01–25. **(H)**. [^18^F]MK-6240 binding to CN TCX 01–25; autoradiography scale bar: 0–1500 DLU/mm^2^. **(I)**. [^18^F]MK-6240 binding in DLU/mm^2^ to all CN cases within GM and WM of TCX and FCX regions. **(J)**. Average [^18^F]MK-6240 binding to all CN cases separated by TCX and FCX GM and WM regions (ns=not significant).

Figure 3 depicts [^18^F]MK-6240 binding in all CN cases. Fig. 3A–D showed adjacent slices of representative case 01–25 CN FCX while Fig. 3E–H showed case 01–25 CN TCX. Anti-tau immunostains (Fig. 3B, 3F) of the five CN cases revealed little tau, confirmed by the tau pixel classifier (Fig. 3C, 3G). The locations of detected anti-tau and the [^18^F]MK-6240 binding on AD TCX autoradiographic image aligned, however, 03–07 had the highest [^18^F]MK-6240 binding amongst all CN cases. The CN cases 03–07 and 17–14 were assigned the highest tangle stage (Table 1) among all CN cases which may be attributed to higher [^18^F]MK-6240 binding (Fig. 3I). The average GM/WM ratio in TCX was 1.9 while FCX had a GM/WM ratio of 2.1 in CN. This higher ratio may be due to the two outlier CN cases. Regardless, all [^18^F]MK-6240 binding values in CN cases were noticeably lower than AD and DSAD. There were no significant differences between GM and WM in FCX and TCX (Fig. 3J).

#### 3.1.4. Comparison of DSAD, AD and CN Cases

**Figure 4:**
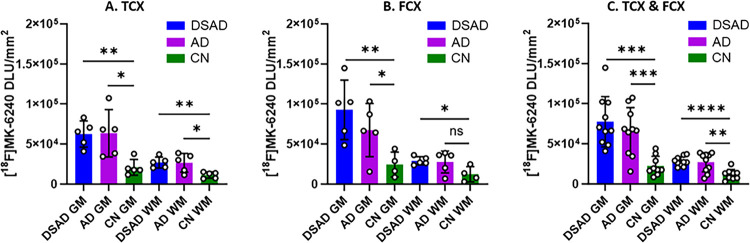
Group Comparisons of [^18^F]MK-6240 Binding: Unpaired two-tailed parametric t-tests determined statistical significance between each parameter (* p < 0.05, ** p < 0.01, *** < 0.001, **** < 0.0001, ns=not significant). **(A)**. [^18^F]MK-6240 binding in TCX GM and WM of DSAD, AD, and CN cases. **(B)**. [^18^F]MK-6240 binding in FCX GM and WM of DSAD, AD, and CN cases. **(C)**. [^18^F]MK-6240 binding in FCX and TCX GM and WM of DSAD, AD, and CN cases.

Figure 4 summarizes the comparisons between DSAD, AD, and CN GM and WM in TCX and FCX [^18^F]MK-6240 binding. As expected, among both TCX (Fig. 4A) and FCX (Fig. 4B) regions, DSAD GM was significantly greater than CN GM. The average WM in both the DSAD and AD cases of TCX (Fig 4A) and FCX (Fig 4B) was higher than the CN cases as well. It is unclear if this higher WM is due to spillover effects of GM or there may be traces of tau within these regions [37, 44]. The GM/WM ratios for each case (DSAD and AD) resulted in no significant differences between TCX and FCX. In consideration of both FCX and TCX regions, the average GM/WM ratios were 2.8, 2.5, and 2.0 for DSAD, AD, and CN cases respectively. The higher average ratio in CN subjects was driven by the two outliers. Although within the CN, the GM/WM was higher than expected, the GM ratio of AD/CN and DSAD/CN were 2.9 and 3.5 respectively, confirming the diagnostic ability of [^18^F]MK-6240 for tau.

### 3.2. In Vitro Binding Inhibition of [^18^F]MK-6240 by Harmine

**Figure 5:**
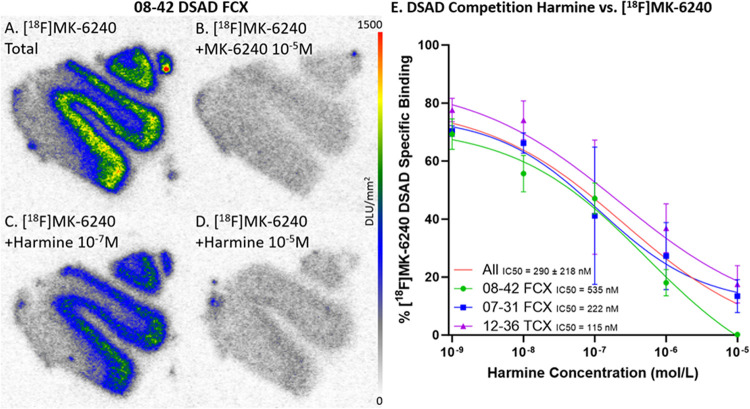
Harmine inhibition on [^18^F]MK-6240 Binding in DSAD: Autoradiography scale bar: 0–1500 DLU/mm^2^. **(A)**. DSAD case 08–42 FCX total [^18^F]MK-6240 binding without harmine. **(B)**. Case 08–42 FCX with [^18^F]MK-6240 plus unlabeled MK-6240 (10^−5^ M). **(C)**. Case 08–42 FCX with [^18^F]MK-6240 plus harmine (10^−7^ M).**(D)**. Case 08–42 FCX [^18^F]MK-6240 plus harmine (10^−5^ M). **(E)**. Average [^18^F]MK-6240 specific binding of each DSAD case throughout all harmine concentrations. The red line represents the best fit line for all cases. Each case is plotted with 3 adjacent brain slices for each concentration.

**Figure 6:**
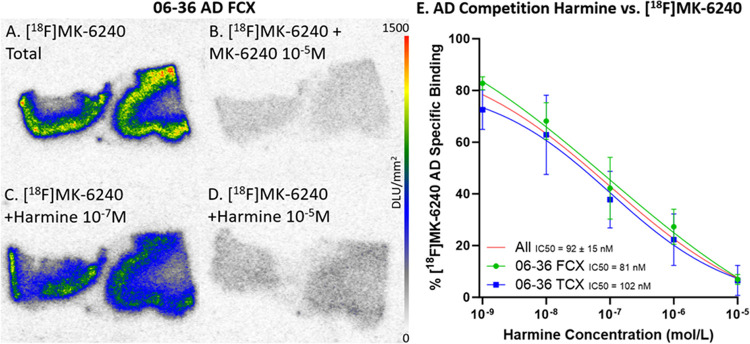
Harmine inhibition on [^18^F]MK-6240 Binding in AD: Autoradiography scale bar: 0–1500 DLU/mm^2^. **(A)**. AD case 06–36 FCX total [^18^F]MK-6240 binding without harmine. **(B)**. Case 06–36 FCX with [^18^F]MK-6240 plus unlabeled MK-6240 (10^−5^ M). **(C)**. Case 06–36 FCX with [^18^F]MK-6240 plus harmine (10^−7^ M). **(D)**. Case 06–36 FCX [^18^F]MK-6240 plus harmine (10^−5^ M). **(E)**. Average [^18^F]MK-6240 specific binding of case 06–36 FCX and TCX throughout all harmine concentrations. The red line represents the best fit line for all brain regions. Each brain region is plotted with 3 adjacent brain slices for each concentration.

Selective in vitro [^18^F]MK-6240 binding patterns were investigated during drug competition studies using harmine which has been shown to bind to MAO-A and DYRK1A [42] (Fig. 5). Per case, there were three adjacent FCX and TCX brain slices within each of the five harmine concentrations. Specific binding was obtained by subtracting nonspecific binding ([^18^F]MK-6240 plus unlabeled MK-6240, Fig. 5B & 6B) from the total binding of [^18^F]MK-6240 (Fig. 5A & 6A). Similarly, specific binding of different [^18^F]MK-6240 + harmine concentrations were calculated (Fig 5C–D & 6C–D). There was greater displacement of [^18^F]MK-6240 as harmine concentrations increased. The competitive effect of harmine was consistent within both TCX and FCX regions and across different DSAD (Fig. 5E) and AD cases (Fig. 6E). The average IC_50_ values for harmine were measured to be IC_50_=290±218 nM and IC_50_=92±15 nM for DSAD and AD cases respectively. This displacement of [^18^F]MK-6240 by harmine in AD cases is consistent with previously reported findings in AD where a single concentration of harmine was used [3].

### 3.3. In Vitro Drug Effects on [^18^F]MK-6240

**Figure 7:**
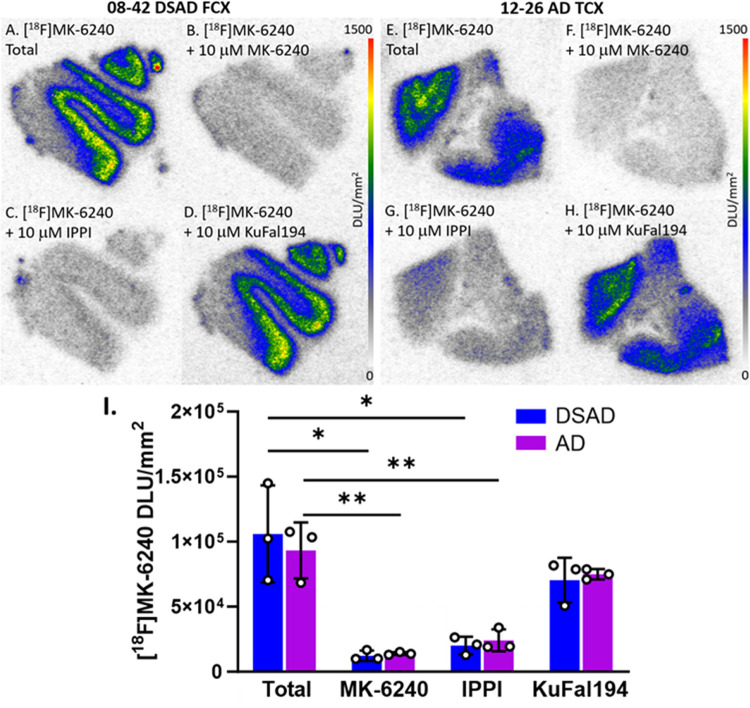
Effect of MK-6240, IPPI, and KuFal194 on [^18^F]MK-6240: Autoradiography scale bar: 0–1500 DLU/mm^2^. **(A)**. DSAD case 08–42 FCX total [^18^F]MK-6240 binding. **(B)**. DSAD case 08–42 FCX [^18^F]MK-6240 plus unlabeled MK-6240 binding. **(C)**. DSAD case 08–42 FCX [^18^F]MK-6240 plus IPPI binding. **(D)**. DSAD case 08–42 FCX [^18^F]MK-6240 plus KuFal194 binding. **(E)**. AD case 12–26 TCX total [^18^F]MK-6240 binding **(F)**. AD case 12–26 TCX [^18^F]MK-6240 plus unlabeled MK-6240 binding. **(G)**. AD case 12–26 TCX [^18^F]MK-6240 plus IPPI binding. **(H)**. AD case 12–26 TCX [^18^F]MK-6240 plus KuFal194 binding. **(I)**. Comparisons between total [^18^F]MK-6240 binding and binding of [^18^F]MK-6240 plus MK-6240, IPPI, and KuFal194 individually within DSAD and AD cases. Unpaired two-tailed parametric t-tests determined statistical significance between each parameter (* p < 0.05, ** p < 0.01).

To further understand how [^18^F]MK-6240 binds in DSAD cases compared to AD cases, various drugs were used to observe their effects on [^18^F]MK-6240 binding in both TCX and FCX (Fig. 7). When compared to total [^18^F]MK-6240 binding (Fig. 7A, 7E), unlabeled MK-6240 and IPPI significantly inhibited [^18^F]MK-6240 binding which was expected and consistent with tau binding (Fig. 7I). Both DSAD and AD exhibited similar displacement in all drug groups. MK-6240 displaced [^18^F]MK-6240 by 88% among DSAD cases and 85% among AD cases. IPPI displaced [^18^F]MK-6240 by 81% and 74% in DSAD and AD cases respectively. KuFal194 minimally displaced [^18^F]MK-6240 by 34% in DSAD cases and 20% in AD cases. Competition of [^18^F]MK-6240 with the DYRK1A inhibitor, KuFal194 exhibited nonsignificant effect, suggesting that [^18^F]MK-6240 does not bind to the phosphokinase, if present, in both AD and DSAD cases.

### 3.4. In Vitro Meninges Binding of [^18^F]MK-6240

**Figure 8:**
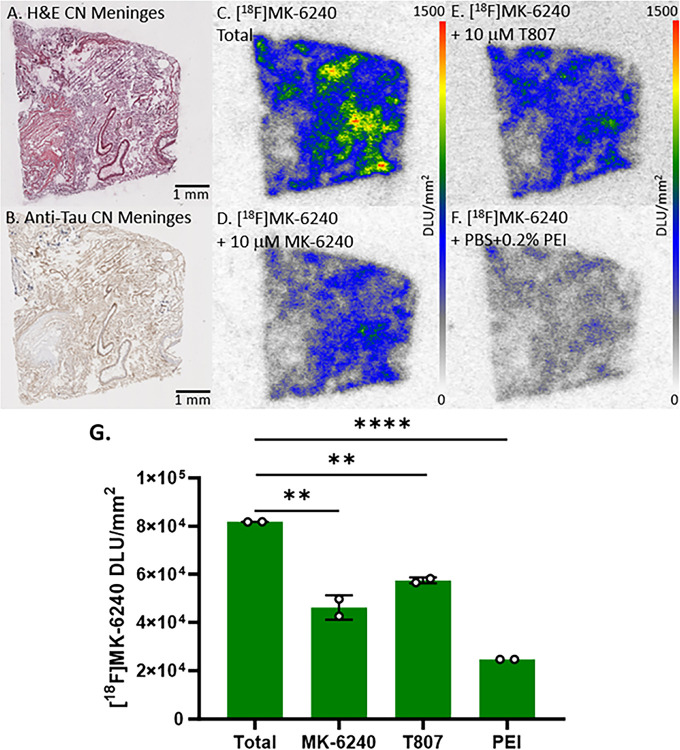
[^18^F]MK-6240 Binding to CN Meninges: Autoradiography scale bar: 0–1500 DLU/mm^2^. **(A)**. H&E IHC of postmortem slice of CN meninges. **(B)**. Anti-tau IHC of CN meninges. **(C)**. [^18^F]MK-6240 total binding to CN meninges. **(D)**. [^18^F]MK-6240 plus 10 μM MK-6240 to CN meninges. **(E)**. [^18^F]MK-6240 binding plus 10 μM T807 to CN meninges. **(F)**. [^18^F]MK-6240 binding with PBS plus 0.2% PEI to CN meninges. **(G)**. Comparison between total [^18^F]MK-6240 binding to binding of [^18^F]MK-6240 plus MK-6240, PEI, and T807 separately. Unpaired two-tailed parametric t-tests determined statistical significance between each parameter (** p < 0.01, **** < 0.0001).

PET studies using [^18^F]MK-6240 in AD subjects have reported off-target binding to the meninges [33, 38]. Understanding the [^18^F]MK-6240 binding characteristics in the meninges is necessary in order to minimize this off-target binding which will assist in the quantification of [^18^F]MK-6240 PET studies. All human meninges postmortem slices for this study were adjacent to one another and from the same CN case. Adjacent meninges slices were immunostained with H&E (Fig. 8A) and anti-tau (Fig. 8B). To quantify [^18^F]MK-6240 binding in the meninges, slices of CN meninges underwent various autoradiography studies. Substantial off-target binding of [^18^F]MK-6240 was confirmed throughout the meninges (Fig. 8C). Compared to total binding, there was self blocking (Fig. 8D) but complete displacement was not achieved with 10 μM MK-6240 (44% decrease). Similarly, the tau imaging agent T807 was not able to block this off-target binding of [^18^F]MK-6240 (30% decrease; Fig. 8E). Because a lack of blocking effect by tau drugs, buffer conditions of incubation of [^18^F]MK-6240 was changed by including PEI. There was a better reduction of [^18^F]MK-6240 (70% decrease, Fig. 8F–G).

**Figure 9:**
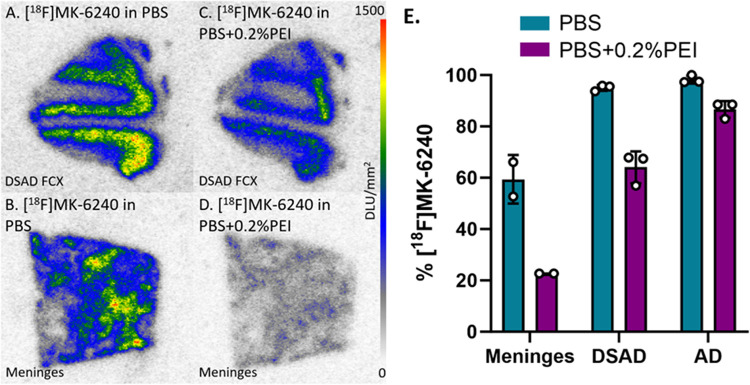
[^18^F]MK-6240 Binding Comparisons with PBS and PEI: Autoradiography scale bar: 0–1500 DLU/mm^2^. **(A)**. [^18^F]MK-6240 binding to DSAD 08–42 FCX with 100% PBS buffer. **(B)**. [^18^F]MK-6240 binding to CN meninges with 100% PBS buffer. **(C)**. [^18^F]MK-6240 binding to DSAD 08–42 FCX with PBS plus 0.2% PEI. **(D)**. [^18^F]MK-6240 binding to CN meninges with PBS and 0.2% PEI. **(E)**. Percent binding of [^18^F]MK-6240 in the presence of PBS or PBS plus 0.2% PEI within CN meninges, DSAD, and AD.

In vitro receptor binding studies have used PEI for purposes of reducing nonspecific binding [9]. Since the presence of PEI resulted in the greatest decrease in [^18^F]MK-6240 binding, there was interest to observe its effect in DSAD and AD cases. Without PEI, the percent binding of [^18^F]MK-6240 to CN meninges was on par with percent binding to DSAD and AD cases (Fig. 9E) while being higher than the average percent binding in other CN cases (Fig. 3). Binding of [^18^F]MK-6240 in PBS containing 0.2% PEI (Fig. 9C–D) was lower than 100% PBS (Fig. 9A–B) in CN meninges, DSAD, and AD. The PBS with 0.2% PEI incubation resulted in a 37%, 31%, and 12% decrease in [^18^F]MK-6240 binding to meninges, DSAD, and AD respectively (Fig. 9E).

### 3.5. In Vitro Meninges Binding of [^125^I]IPPI

**Figure 10:**
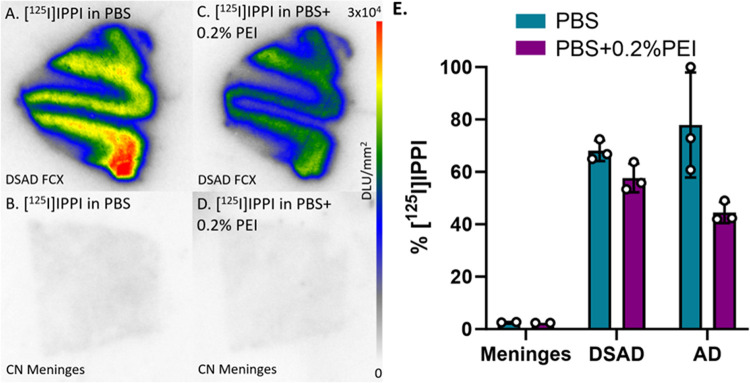
[^125^I]IPPI Binding Comparisons with PBS and PEI: Autoradiography scale bar: 0–30000 DLU/mm^2^. **(A)**. [^125^I]IPPI binding to DSAD 08–42 FCX with 100% PBS buffer. **(B)**. [^125^I]IPPI binding to CN meninges with 100% PBS buffer. **(C)**. [^125^I]IPPI binding to DSAD 08–42 FCX with PBS plus 0.2% PEI. **(D)**. [^125^I]IPPI binding to CN meninges with PBS plus 0.2% PEI. **(E)**. Percent binding of [^125^I]IPPI in the presence of PBS or PBS plus 0.2% PEI within CN meninges, DSAD, and AD.

The [^18^F]MK-6240 analog [^125^I]IPPI was also studied for in vitro binding to meninges to compare with DSAD and AD cases. [^125^I]IPPI exhibited little to no binding in the meninges under the same conditions as [^18^F]MK-6240 (Fig. 10). Meninges binding is comparable to the percent binding in other CN cases where they are significantly lower than DSAD and AD cases (Fig. 10E). The PBS plus 0.2% PEI incubation resulted in a 0.2%, 10%, and 33% decrease in [^125^I]IPPI binding to meninges, DSAD, and AD respectively (Fig. 10E). The percent binding to meninges is already minimal so the effect of the PBS with 0.2% PEI incubation is indistinguishable. Thus, the pattern of [^125^I]IPPI and [^18^F]MK-6240 binding in the meninges were found to be very different.

## 4. Discussion

Several novel radiotracers have been developed to successfully detect tau pathology in human AD, revolutionizing clinical diagnostic strategies. With the genetic predisposition of people with DS developing AD diagnosis, expanding upon this success in DSAD can provide more information about the features and progression of the disease. In this study, [^18^F]MK-6240 binding to tau was validated to be highly selective in both DSAD and AD cases within postmortem human brain slices of FCX and TCX GM. Significant differences were more prominent between DSAD and CN than AD and CN cases. In both DSAD and AD cases, [^18^F]MK-6240 was significantly displaced by harmine, unlabeled MK-6240, and IPPI but not KuFal194. The meninges of a CN case were confirmed to have [^18^F]MK-6240 binding that was significantly self-displaced by 44% despite the minimal tau detected throughout the meninges slice.

As expected, binding of [^18^F]MK-6240 was successful to tau in DSAD and AD cases in FCX and TCX GM. The results in this study similarly match the binding distribution of [^3^H]MK-6240 to tau deposits in postmortem brain slices of human hippocampus and medial frontal gyrus [27]. With both [^3^H]MK-6240 and [^18^F]MK-6240 binding to tau in human DSAD brains, this provides valuable insight into the capabilities of MK-6240 beyond only AD. When comparing FCX and TCX regions, there were minimal differences in [^18^F]MK-6240 binding to tau. Previous literature suggests slight preferential binding to TCX as the expected pattern of tau accumulation develops early in the temporal network [6, 54]. Since all the DSAD and AD cases in this study were Braak stage IV, there is no clear distinction between FCX and TCX tau accumulation that may differ in ascending Braak stages. In DSAD stages III-VI, tau pathology appears more prevalent in the FCX [27]. Regardless, these results validate the potential in using [^18^F]MK-6240 as a reliable tau imaging agent in both DSAD FCX and TCX.

Based on the competition curves, [^18^F]MK-6240 binding was inhibited by increasing concentrations of harmine with each case sharing similar IC_50_s. This is similar to our previous observation on the effects of harmine on [^125^I]IPPI [23]. Harmine binds to multiple forms of phosphorylated tau including total tau which is abundant in all DSAD and AD cases [53]. However, harmine may not be the most informational agent for tau since it binds to not only phosphorylated tau but also to DYRK1A and MAO-A, therefore hindering the binding specificity [53]. DYRK1A inhibition has previously been shown to improve cognitive function in DS but the link between DYRK1A and tau is only established in postmortem human tissue [18, 32]. DYRK1A inhibitors are a potential therapeutic strategy to prevent tau hyperphosphorylation so determining compounds that inhibit DYRK1A can attest to this strategy [14, 42]. However, the DYRK1A agent KuFal194 did not substantially inhibit [^18^F]MK-6240 binding among DSAD and AD cases. Similarly, the MAO-A inhibitor clorgyline did not inhibit [^18^F]MK-6240 binding, so harmine by inference seems to uniquely compete with tau agents at the tau binding site [3]. Thus, [^18^F]MK-6240 binding appears selective to tau and not to MAO-A sites or to DYRK1A sites in the brain slices. Similar findings of tau selectivity were reported for [^125^I]IPPI which is an analog of MK-6240 [23].

To accurately assess tau accumulation, tracking and minimizing off-target binding is ideal. Although [^18^F]MK-6240 has been demonstrated to be selective for tau, ongoing PET studies in human subjects have identified a significant amount of nonspecific off-target binding to the meninges [33, 38]. Accurate quantification of cortical [^18^F]MK-6240 in AD subjects therefore requires careful separation from any meninges spillover effects [33, 38]. Our postmortem meninges tissue evaluation revealed extensive [^18^F]MK-6240 binding which was nonspecific off-target since there was an absence of tau confirmed by immunostains. A majority of this binding in the meninges was therefore not displaceable by tau agents MK-6240 and T807. The cationic polymer PEI inhibited [^18^F]MK-6240 binding the most. Nonspecific binding has been minimized by using up to 1% PEI with carbon-11 and fluorine-18 labeled radioligands binding to cloned receptors and human AD brain tissue [9, 11–12, 26]. Electrostatic interactions of PEI with negatively charged cell surfaces may assist specific binding between ligands and receptors including in the cerebral meninges [24, 29]. The dural lymphatic system importantly facilitates the clearance of extracellular tau so its dysfunction results in tau deposition and therefore promoting neurodegeneration [43, 48]. Meningeal lymphatic vessels are crucial for fluid drainage but exhibit a decrease in function with increasing age [16, 25]. The CN case used for meninges was 66, an age that is at risk of dysfunction of the lymphatic system. However, there was little to no tau detected via anti-tau staining on an adjacent slice. Although [^18^F]MK-6240 is not necessarily binding to tau in these meninges, the binding to the cells containing melanin may be nonspecific [2]. Recent literature reveals in vivo PET/MRI variability in magnitude and distribution of [^18^F]MK-6240 binding to meninges in CN cases [33]. Interestingly, [^18^F]MK-6240 PET SUVR showed heterogeneity in extra-cerebral off-target binding to meninges in different cases and inter-case scans [38].

**Figure 11:**
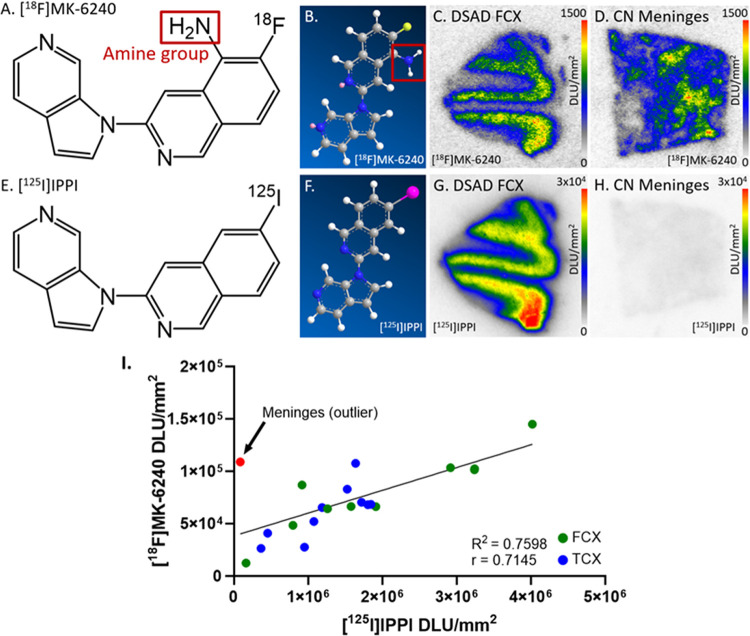
Comparison of [^18^F]MK-6240 and [^125^I]IPPI structure and binding: **(A)**. Chemical structure of [^18^F]MK-6240. Red outline indicates location of amine group. **(B)**. Ball-and-stick structure of [^18^F]MK-6240. Red outline indicates location of amine group. **(C)**. [^18^F]MK-6240 binding to DSAD FCX slice (10 μm); autoradiography scale bar: 0–1500 DLU/mm^2^. **(D)**. [^18^F]MK-6240 binding to CN meninges slice (10 μm); autoradiography scale bar: 0–1500 DLU/mm^2^. **(E)**. Chemical structure of [^125^I]IPPI. **(F)**. Ball-and-stick structure of [^125^I]IPPI. **(G)**. [^125^I]IPPI binding to DSAD FCX slice (10 μm); autoradiography scale bar: 0–30000 DLU/mm^2^. **(H)**. [^125^I]IPPI binding to CN meninges slice (10 μm); autoradiography scale bar: 0–30000 DLU/mm^2^. **(I)**. Correlation between [^125^I]IPPI and [^18^F]MK-6240 binding to FCX and TCX regions of DSAD and AD cases (Pearson’s *r*=0.7145; R^2^=0.7598). Average meninges binding was included to visualize on the correlation plot but was not included in the Pearson’s correlation.

To further ascertain this meninges binding, we used [^125^I]IPPI, which is an analog for [^18^F]MK-6240. There was little to no [^125^I]IPPI binding in meninges while [^18^F]MK-6240 binds to meninges in a comparable amount to DSAD FCX (Fig. 11C–D, 11H). The primary structural difference between the two radiotracers is the amine group on [^18^F]MK-6240 that is not present on [^125^I]IPPI (Fig. 11A, 11E). Both radiotracers bind to DSAD and AD FCX and TCX regions similarly while there is a major difference in meninges binding (Fig. 11I) The unique features of [^18^F]MK-6240 off-target binding must continue to be investigated to maximize accuracy in [^18^F]MK-6240 binding throughout the brain. Along these lines, it may be of value to study the [^18^F]analog previously reported alongside [^18^F]MK-6240, which lacks the amino group and is more similar to [^125^I]IPPI (^125^I- replaced with ^18^F- [56]).

Despite the valuable findings of this study, there are also limitations. There is a small number of DSAD, AD, and CN cases categorized in the same Braak stage, offering minimal diversity in tau abundance and disease severity. Some inter-case variability within adjacent brain slices was present but did not affect the acquisition of [^18^F]MK-6240 binding. Cases were not age matched between different groups, so CN cases were the oldest while DSAD and AD cases were more similar in age. More cases of meninges in order to further confirm results from this study would be useful. Nevertheless, this study still establishes the use of [^18^F]MK-6240 as a radioligand to elucidate tau accumulation in DSAD while future studies will aim to improve upon the listed limitations.

In conclusion, the autoradiography binding features of [^18^F]MK-6240 hold promise in its application in FCX and TCX regions in DSAD brains similarly to AD tauopathy. Current AD strategies can be applied to DSAD as an initial basis and then adapted to best approach unique features of DSAD. Further studies can validate novel applications based on the intended use of selective radiotracers such as [^18^F]MK-6240, highlighting more of their capabilities in detecting tau aggregation.

## Figures and Tables

**Table 1:** Case tissue samples and data.

Case ID^1^	Pathology	Gender	Age At Death	PMI^2^	Braak Score^3^	Plaque Stage^4^	Tangle Stage^5^
01–25	CN	Male	83	1.8	II	0	2
02–11	CN	Male	96	3.58	II	0	2
03–07	CN	Female	84	4.25	III	A	3
12 – 09	CN	Female	95	2.92	II	0	2
17 – 14	CN	Female	89	5.95	III	0	3
06–18	AD	Male	46	3	VI	C	6
06–36	AD	Male	61	5.58	VI	C	6
12–26	AD	Male	55	3.18	VI	C	6
09 – 05	AD	Female	57	3.17	VI	C	6
16–38	AD	Female	76	3.82	VI	B	6
11–30	DSAD	Male	66	4.08	VI	C	6
08–42	DSAD	Male	55	4.5	VI	C	6
10–31	DSAD	Female	62	2.42	VI	C	6
07–31	DSAD	Female	52	4.37	VI	C	6
12–36	DSAD	Female	56	4.08	VI	C	6

Frozen brain tissue samples of FCX and TCX were obtained from UCI MIND Institute; CN = cognitively normal; AD = Alzheimer’s disease; DSAD = Down syndrome-associated Alzheimer’s disease.

1 Each autopsy case identified by the year and order of autopsy (e.g. 08–42 is the 42nd autopsy performed in 2008).

2 PMI: Postmortem interval in hours.

3 Braak staging reflects pTau181 burden.

4 Plaque stage: Includes neuritic, cored and diffuse. Semi-quantitative scores of none, sparse, moderate and frequent were converted to a Plaque stage of A – C.

5 Tangle stage: neurofibrillary tangle density indicated by numerical values 0–6 for Tangle stage.

## Data Availability

The data that support the findings of this study are available from the corresponding author upon reasonable request.
